# Efficacy of Azamethiphos for Grain and Surface Treatment Against Three Major Stored-Product Beetle Species

**DOI:** 10.3390/insects17080820

**Published:** 2026-08-07

**Authors:** Maria K. Sakka, Eirini Karanastasi, Reggina Krassi, Marianna Rigopoulou, Ioannis P. Markou, Georgia Baliota, Paraskevi Agrafioti, Christos G. Athanassiou, Christos I. Rumbos

**Affiliations:** 1 Laboratory of Entomology and Agricultural Zoology, Department of Agriculture, Crop Production and Rural Environment, University of Thessaly, Phytokou Str., 38446 Volos, Greece; msakka@uth.gr (M.K.S.); mrigopoulou@uth.gr (M.R.); mpaliota@uth.gr (G.B.); agrafiot@uth.gr (P.A.); athanassiou@uth.gr (C.G.A.); 2 Laboratory of Plant Protection, Department of Agriculture, University of Patras, Messolonghi University Campus, 30200 Messolonghi, Greece; ekaranastasi@upatras.gr (E.K.); up1076828@ac.upatras.gr (R.K.); i.markou@ac.upatras.gr (I.P.M.); 3 Laboratory of Entomology, Department of Agriculture, International Hellenic University, Alexander Campus, 57400 Thessaloniki, Greece

**Keywords:** grains, surface treatment, concrete, plastic, mortality, beetles

## Abstract

The present study evaluated the insecticidal efficacy of a commercial formulation containing azamethiphos (Teenox^®^ EC) against adults of the saw-toothed grain beetle, *Oryzaephilus surinamensis*, the lesser grain borer, *Rhyzopertha dominica* (F.), and the granary weevil, *Sitophilus granarius* (L.), under laboratory conditions. The azamethiphos formulation was assessed both as grain treatment on wheat and barley at 0, 2.5, 5, 10 and 20 ppm for all species, and as surface treatment on concrete and plastic surfaces at 0, 0.125, 0.25, 0.5 and 1 μg a.i./cm^2^ for *O. surinamensis* and *R. dominica*. In grain treatments, adult mortality increased significantly with concentration and exposure interval for all species. Mortality was generally higher in barley than in wheat, particularly after longer exposure periods. *Sitophilus granarius* was the most susceptible species, reaching high mortality at the highest concentration, whereas *R. dominica* exhibited lower mortality responses, especially at lower concentration. Progeny production was significantly reduced with increasing concentration for all species, although the effect was more pronounced in barley than in wheat, particularly for *O. surinamensis*. In surface treatments, mortality was strongly influenced by surface type, with significantly higher efficacy on concrete compared to plastic. Mortality increased with both concentrations and exposure time for all species, while interspecific differences were less pronounced than in grain treatments. These responses indicate that both substrate characteristics and exposure time are important parameters of azamethiphos efficacy. Overall, the results indicate that the azamethiphos formulation tested can provide effective control of key stored-product beetle species when applied either on grains or different surfaces, although its performance is influenced by commodity and surface type. These findings highlight the potential of azamethiphos as a versatile tool in integrated stored-product pest management programs.

## Simple Summary

Stored-product insects are responsible for significant quantitative and qualitative losses in stored cereals worldwide. Chemical insecticides remain an important tool for controlling these pests both as grain protectants and as structural surface treatments in storage facilities. In the present study, we evaluated the insecticidal efficacy of a commercial formulation containing azamethiphos (Teenox^®^ EC) against three major stored-product beetle species, the saw-toothed grain beetle, *Oryzaephilus surinamensis* (L.), the lesser grain borer, *dominica* (F.) and the granary weevil, *Sitophilus granarius* (L.). The formulation was tested both as grain treatment on wheat and barley and as surface treatment on concrete and plastic surfaces. Mortality increased with increasing concentration and exposure intervals, while progeny production was significantly reduced at higher concentrations. In general, higher mortality levels were recorded in barley than in wheat, whereas surface treatments were more effective on concrete compared with plastic. These findings indicate that the tested azamethiphos formulation could represent a useful tool for the management of stored-product insects within integrated pest management programs.

## 1. Introduction

Stored-product insects are among the most important pests affecting the quality and quantity of stored agricultural commodities causing post-harvest losses that may reach up to 20% worldwide [1,2]. Infestations by these insect species may result in significant economic losses due to direct feeding damage, product weight loss and contamination with insect body fragments, exuviae and metabolic byproducts, which can significantly reduce the commercial value of the stored commodities [1,3,4,5]. Except for the direct product damage, infestations by stored-product insects may change the composition and degrade the products’ quality leading also to toxicity and safety concerns; they facilitate the development of microorganisms, particularly fungi associated with mycotoxin production and further contribute to deterioration and contamination of stored products [4,6,7]. Consequently, stored-product insect management remains a major component of post-harvest protection systems worldwide.

Among stored-product insects, the saw-toothed grain beetle, *Oryzaephilus surinamensis* (L.) (Coleoptera: Silvanidae), is one of the most widespread and economically important secondary pests of stored commodities. Despite being a secondary pest, it can cause significant economic losses through contamination and deterioration at high population densities [8]. It is highly polyphagous, infesting more than 140 products, including cereals, processed foods and dried fruit [9,10,11,12] and is frequently found in storage facilities and food-processing environments even in the absence of food residues [13,14]. In addition, the lesser grain borer, *Rhyzopertha dominica* (F.) (Coleoptera: Bostrychidae), and the granary weevil, *Sitophilus granarius* (L.) (Coleoptera: Curculionidae), are major primary pests of stored cereals. Both species cause substantial grain damage, weight loss and quality degradation, and are widely distributed in storage facilities worldwide [1,9,14,15].

The management of stored-product insects relies on a combination of preventive and curative measures including sanitation, monitoring and chemical control. Despite the increasing interest in alternative control strategies, conventional insecticides remain one of the most widely used tools for protecting stored commodities and facilities from insect infestations and remain difficult to completely replace [16,17,18]. Insecticides may be applied either directly to the stored commodity as grain protectants or on structural surfaces such as floors, walls and equipment to prevent insect establishment and population growth [9,16,19]. Surface treatments are particularly important because many stored-product insects utilize structural surfaces as refuges and movement pathways within storage facilities [15,20].

The efficacy of insecticides used in stored-product protection may vary considerably depending on several factors, including the insect species, exposure duration, application rate and the characteristics of the treated substrate. Previous studies have demonstrated that surface type may significantly influence the residual efficacy of contact insecticides due to differences in porosity, absorption capacity and insecticide persistence [5,17,21,22]. In addition, the type of treated commodity may affect the insecticide performance and residual activity and consequently influence insect mortality and population development [23,24]. For this reason, evaluation of an insecticide’s performance on different substrates, grains or surfaces, both commonly encountered in storage environments is essential for the determination of their practical applicability, lower cost or capability for long-term results in stored-product protection programs [18,25,26,27].

Azamethiphos is an organophosphate insecticide that acts by inhibiting acetylcholinesterase, thereby disrupting nerve impulse transmission and ultimately causing paralysis and death in insects [28]. Organophosphate insecticides have historically been widely used in pest management programs due to their rapid contact toxicity and broad-spectrum activity against insect pests [29]. Previous studies have reported that azamethiphos can exhibit substantial residual activity when applied on storage surfaces, although its efficacy may vary depending on surface characteristics and environmental conditions [22,29].

Although azamethiphos has been tested against stored-product psocids [22,29,30] limited information is available on stored-product beetles. For instance, Nayak et al. [29] reported that azamethiphos treatments provided long-term control of the psocid *Liposcelis decolor* (Pearman) on galvanized steel surfaces, whereas they failed to provide long-term control on concrete surfaces, indicating that surface type can strongly affect performance. Moreover, data on its performance as both a grain protectant and structural surface treatment remain scarce. The present study aimed to evaluate the insecticidal efficacy of a commercial formulation containing azamethiphos against adults of *O. surinamensis*, *R. dominica* and *S. granarius* under laboratory conditions. Specifically, the formulation was evaluated both as grain treatment on wheat and barley kernels and as surface treatment on two common storage surfaces, concrete and plastic. For this purpose, adult mortality was recorded at different exposure intervals, while progeny production was assessed in the grain-treatment bioassays in order to determine the potential of the tested formulation to suppress population development of these important stored-product pests.

## 2. Materials and Methods

### 2.1. Insects and Insecticide Formulation

Individuals of *O. surinamensis*, *R. dominica* and *S. granarius* from the rearings maintained at the Department of Agriculture in the University of Patras were used for experimentation. Insect rearings were maintained at 25 ± 1 °C, 65% relative humidity (r.h.) and continuous darkness. *Oryzaephilus surinamensis* individuals were reared on oats, whereas *R. dominica* and *S. granarius* on soft wheat kernels. Adult beetles, <1 month-old, were used in the bioassays.

A commercial insecticide formulation (Teenox^®^ EC, Armosa Tech S.A. Engis, Belgium), containing the active ingredient azamethiphos (10% *w*/*v*), was used in all bioassays. The formulation was applied at different concentrations depending on the type of bioassay. For the grain-treatment bioassays, the formulation was applied at 0, 2.5, 5, 10, and 20 ppm (mg of active ingredient (a.i.) per kg of grain). For the surface treatment bioassays, the concentrations applied were 0.125, 0.25, 0.5 and 1 μg a.i./cm^2^.

### 2.2. Bioassay I: Grain Treatment

In a first series of bioassays, the azamethiphos formulation was tested as grain treatment against *O. surinamensis*, *R. dominica* and *S. granarius*. The tests were carried out on intact wheat and barley kernels. The moisture content of wheat was adjusted to 13.5% with a moisture meter (Multitest, Gode SAS, Le Catelet, France). The following concentrations were applied, 0, 2.5, 5, 10 and 20 ppm, while untreated grains served as the control. Briefly, lots of wheat or barley (500 g/replicate/concentration) were placed in 1 L glass jars and sprayed with the azamethiphos formulation at the four concentrations, i.e., 2.5, 5, 10 and 20 ppm (mg a.i./kg of grains). The required azamethiphos solution was applied at a volume of 1 mL of solution was sprayed onto each 500 g grain lot. One additional series of lots was sprayed with distilled water and served as controls. and served as control (0 ppm). The jars have afterwards been shaken manually for approximately 2 min to achieve an equal distribution of the formulation in the entire wheat mass. From each jar, 20 g subsamples were taken and placed in cylindrical plastic vials (3 cm in diameter, 8 cm in height; Rotilabo^®^ sample tins with snap-on lid, Carl Roth, Karlsruhe, Germany). The vials have a plastic lid with a hole in the center covered with fine mesh, while the internal “neck” of the vials was covered by Fluon (Northern Products, Woonsocket, RI, USA), to prevent individuals from escaping. For all beetles, twenty individuals were placed within each vial (separate vials for each species), and the vials were kept in incubators (Elvem, Spata, Greece) set at 25 °C and 65% relative humidity. After 3 d, all the vials were opened and adult mortality were recorded. Dead adults were removed from the vials. Then, the vials were placed back in the incubators. The same procedure was repeated after 7, 14 and 21 d. After the final mortality evaluation, all adults (dead and alive) were removed from the vials and the vials were placed back in the incubators, at the same conditions, for an additional period of 65 d. After the termination of this interval, the vials were opened, and the number of (dead and alive) progeny was recorded. The entire procedure was replicated three times by preparing new lots of treated and untreated grains with each replicate, and exposing, counting, and recording data for mortality and progeny as described above (3 × 3 vials for each concentration × species combination).

### 2.3. Bioassay II: Surface Treatment

In the second series of bioassays, the azamethiphos formulation was tested as surface treatment against both abovementioned insect species. The following were applied: 0.125, 0.25, 0.5 and 1 μg a.i./cm^2^, while untreated surfaces served as the control (0 μg a.i./cm^2^) was sprayed with distilled water. For the surface treatments, 1 mL of the corresponding azamethiphos solution was applied evenly to each 90 mm Petri dish.

The experiments were carried out in standard plastic 90 mm diameter Petri dishes (SARSTEDT AG & Co KG, Nümbrecht-Rommelsdorf, Germany; 62 cm^2^ bottom surface) with bottoms covered with cement or uncovered, as in the case of plastic. Concrete was purchased from a local hardware store (Gray concrete 42.5, Isomat S.A., Thessaloniki, Greece). To prepare the dishes covered with concrete, concrete was mixed with tap water at a ratio of 4:1 and stirred thoroughly to a thick but easy to handle paste. Around 15 mL of this paste was poured into each Petri dish to create an approximately 40 mm thick concrete bottom, which was left to harden and dry for 24 h. The Petri dish walls were covered by Fluon (Northern Products, RI, USA) to prevent insects from escaping. In each bioassay, twenty adults of mixed sexes, randomly collected from the laboratory cultures, were placed in each dish, with separate dishes for each species. As a food source, approximately 5 kernels of cracked wheat were added into each dish. There were 3 dishes for each insect species–concentration–surface type combination. All dishes were kept in an incubator at 25 °C, 65% relative humidity, and continuous darkness. Insect mortality, determined by the absence of movement when probed with a paint brush, was assessed after 3, 7, 14 and 21 d of exposure for all species using a stereomicroscope (Leica KL300 LED, Leica Microsystems, Wetzlar, Germany). The same procedure (three-dishes replicates) was repeated three times (three series of dishes) by preparing new treated and control dishes each time (3 × 3 dishes for each treatment).

### 2.4. Statistical Analysis

To assess differences in mortality of the tested insect species across exposure periods, the data were first analyzed using multivariate analysis of variance (MANOVA), with time interval (days of exposure) as the repeated-measures factor, percent insect mortality as the response variable, and insect species, commodity, and azamethiphos concentration as the main effects. Mortality means among concentrations within each insect species, commodity, and exposure day were then compared using Tukey’s HSD post hoc tests at α < 0.05. The same procedure was followed for the corresponding surface treatments. Progeny counts were first analyzed using a Poisson GLM with insect species, concentration, commodity, and their interactions. The analysis indicated substantial overdispersion (Pearson χ^2^/df = 23.16). Therefore, progeny counts were analyzed using a negative binomial model to account for overdispersion. Progeny means among concentrations within each insect species and commodity were then compared using HSD post hoc tests at α < 0.05.

## 3. Results

### 3.1. Grain Treatments

Mortality differed significantly by commodity type and concentration in all of the three insect species tested (Table 1). In addition, significant differences were also recorded between commodity type and concentration within each insect species (Table 1).

Time of exposure also had a significant effect on mortality within each insect species, and all interactions involving time were significant (*p* < 0.0001), indicating that mortality levels increased over time and differed among insect species, commodities, and concentration (Table 1).

**Figure 1:**
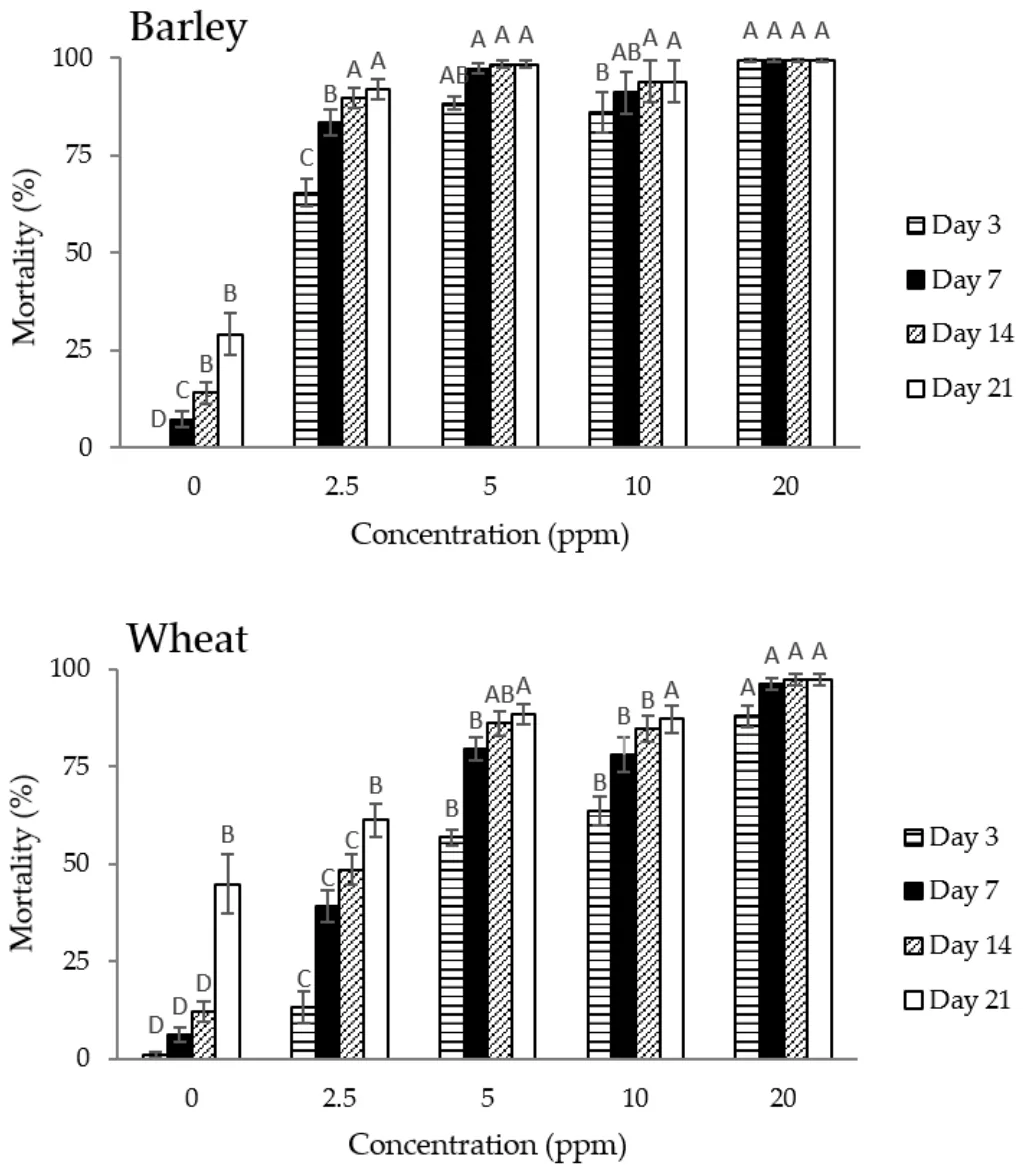
Mean mortality (% ± SE) of *Oryzaephilus surinamensis* after 3, 7, 14 and 21 days of exposure to barley (**upper graph**) or wheat (**lower graph**) treated with five concentrations (0, 2.5, 5, 10 and 20 ppm) of an azamethiphos formulation (Teenox^®^). Different letters among concentrations within each day of exposure indicate significant differences according to HSD at α < 0.05.

**Figure 2:**
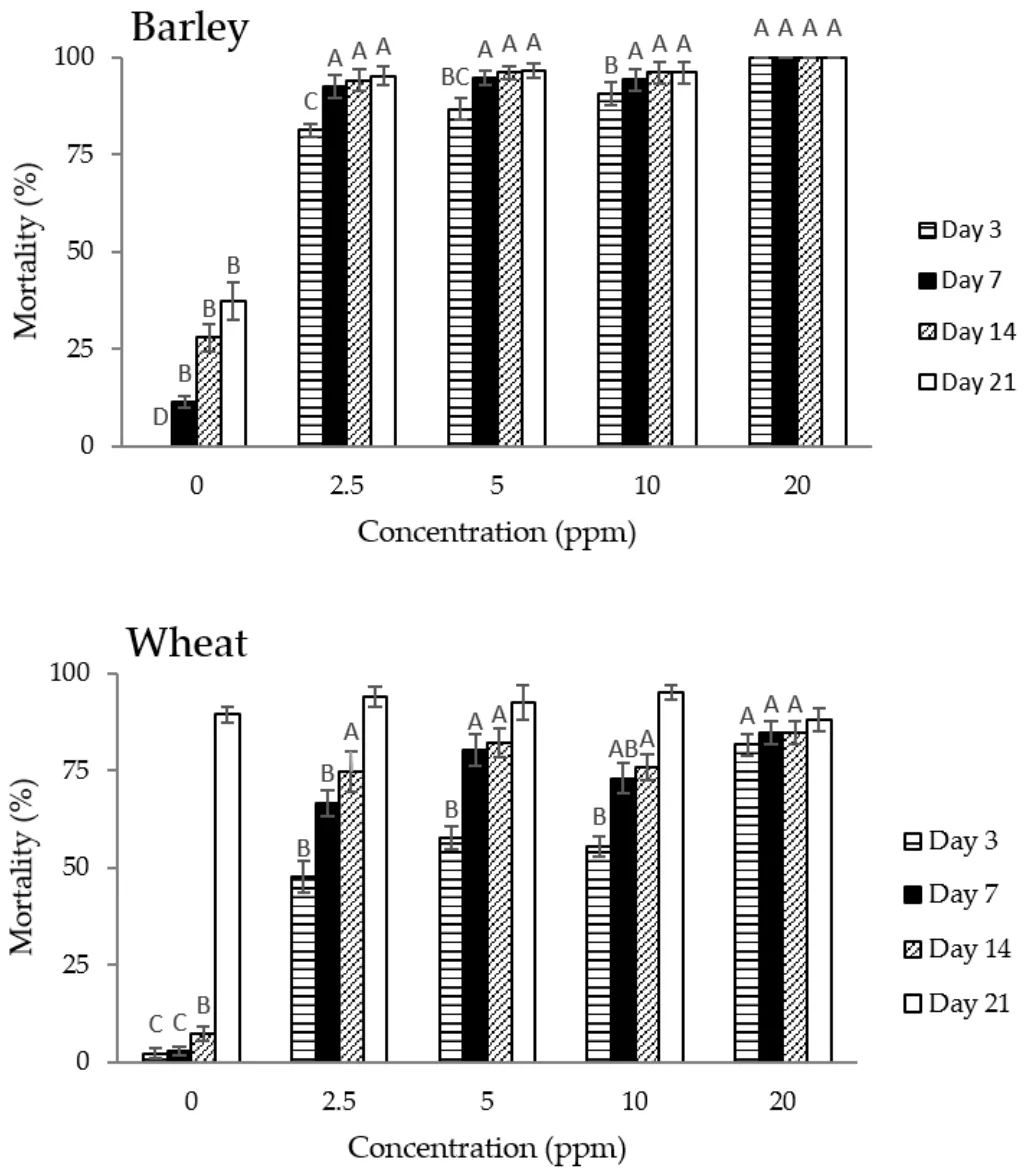
Mean mortality (% ± SE) of *Rhyzopertha dominica* after 3, 7, 14, and 21 days of exposure to barley (**upper graph**) or wheat (**lower graph**) treated with five concentrations (0, 2.5, 5, 10 and 20 ppm) of an azamethiphos formulation (Teenox^®^). Different letters among concentrations within each day of exposure indicate significant differences according to HSD at α < 0.05. Where no letters exist, no significant differences were found.

**Figure 3:**
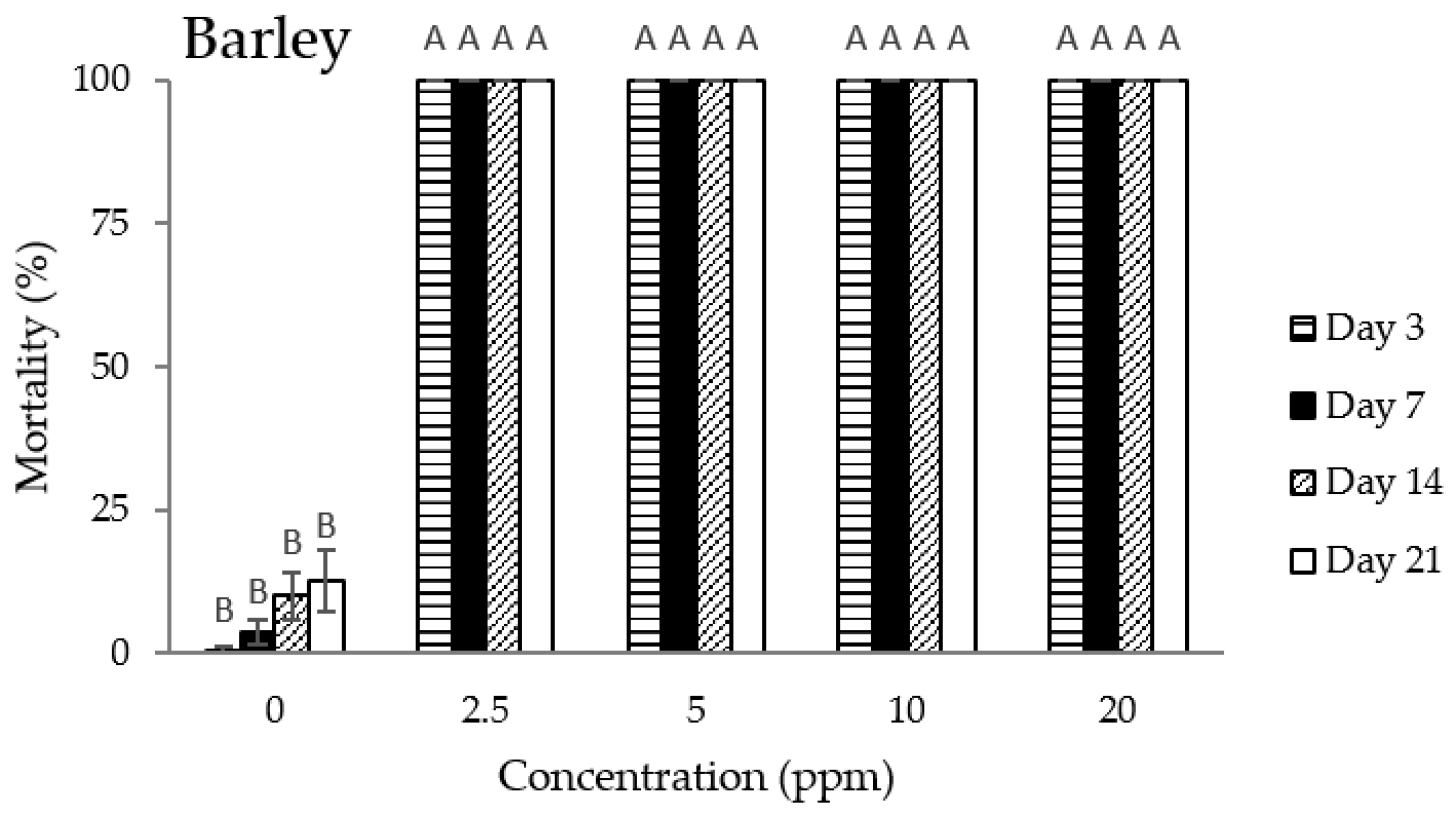

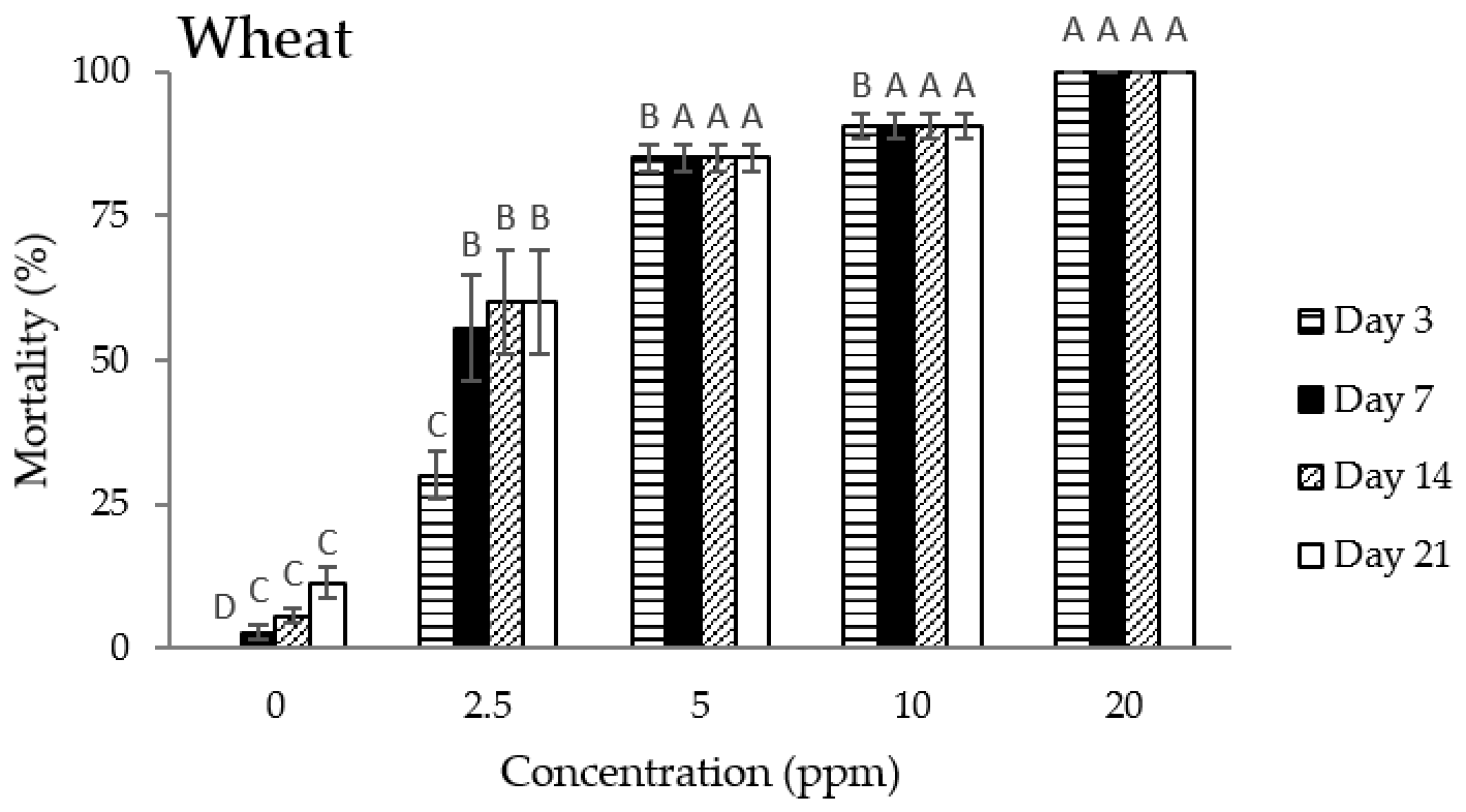
Mean mortality (% ± SE) of *Sitophilus granarius* after 3, 7, 14, and 21 days of exposure to barley (**upper graph**) or wheat (**lower graph**) treated with five concentrations (0, 2.5, 5, 10 and 20 ppm) of an azamethiphos formulation (Teenox^®^). Different letters among concentrations within each day of exposure indicate significant differences according to HSD at α < 0.05.

For *O. surinamensis*, mortality increased with both concentrations and exposure interval in both commodities (Figure 1). At the highest concentration, mortality was significantly higher than the control and lower concentration from early exposure intervals, whereas differences among intermediate concentrations became more pronounced with increasing exposure time. In general, mortality levels were higher in barley than in wheat, particularly at longer exposure periods. Similar trends were observed for *R. dominica* (Figure 2), in which mortality increased significantly with increasing concentration and exposure interval. However, the response of this species was more variable, with fewer significant differences among lower concentration, especially at shorter exposure intervals. For *S. granarius*, mortality also increased with concentration and time (Figure 3), with the highest concentration resulting in almost complete mortality at later exposure intervals. Differences among concentrations were significant for most exposure periods, indicating a strong concentration-dependent response.

### 3.2. Progeny Production

Progeny production was significantly affected by commodity type and concentration but only in the case of *R. dominica* and *S. granarius*, whereas no significant differences were observed by the commodity alone in the case of *O. surinamensis* (Table 2). Significant differences were recorded between commodity type and concentration, regardless of the insect species (Table 2).

**Figure 4:**
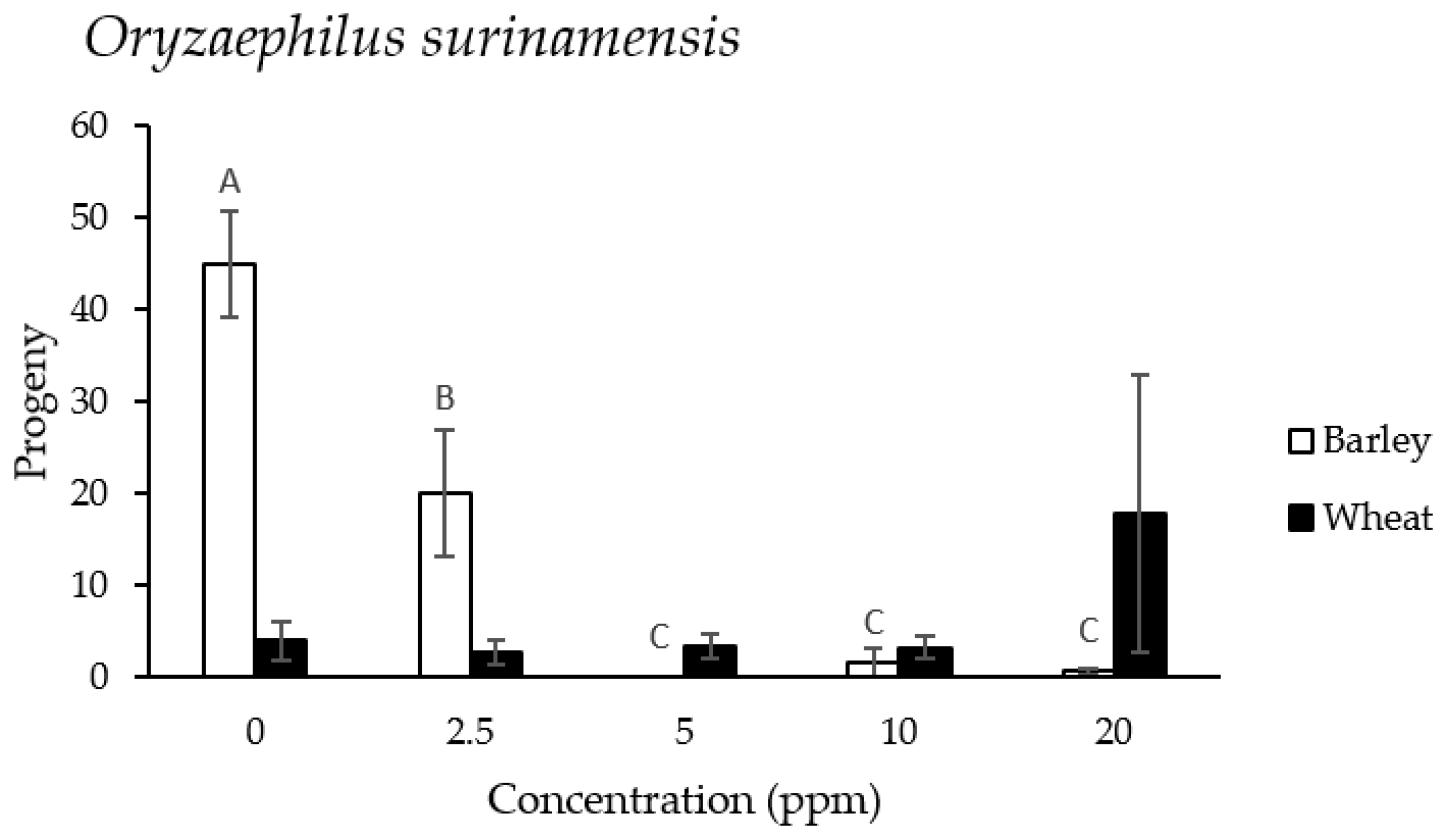
Mean progeny (number of individuals ± SE) of *Oryzaephilus surinamensis* after 65 days of exposure to barley or wheat treated with five concentrations (0, 2.5, 5, 10 and 20 ppm) of an azamethiphos formulation (Teenox^®^). Different uppercase letters among concentration within barley indicate significant differences according to Tukey’s HSD at α < 0.05. No significant differences were found in the progeny production among the concentrations for wheat treatments.

**Figure 5:**
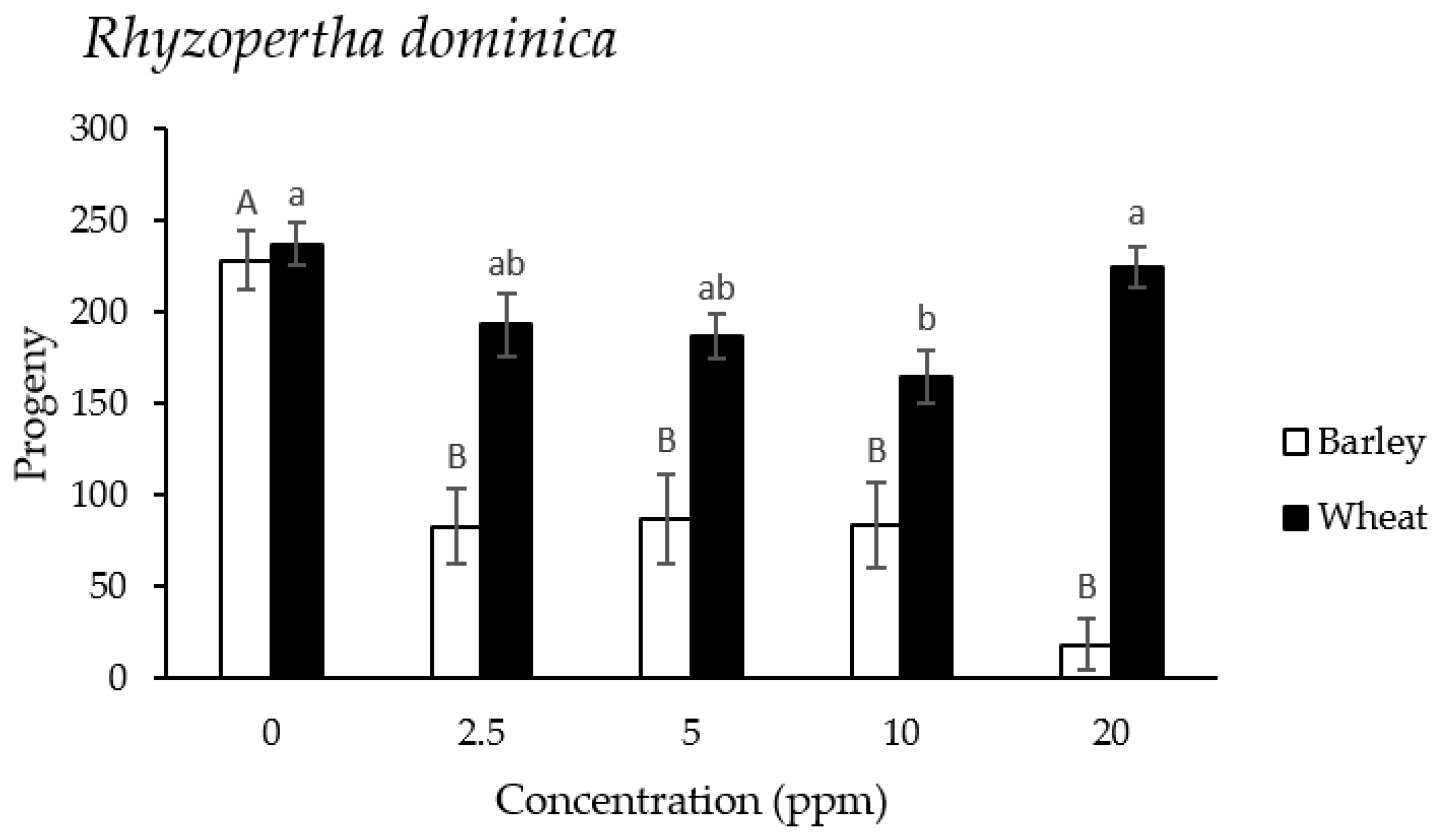
Mean progeny (number of individuals ± SE) of *Rhyzopertha dominica* after 65 days of exposure to barley or wheat treated with five concentrations (0, 2.5, 5, 10 and 20 ppm) of an azamethiphos formulation (Teenox^®^). Different uppercase letters among the concentrations within barley and different lowercase letters among the concentrations within wheat commodity indicate significant differences according to Tukey’s HSD at α < 0.05.

**Figure 6:**
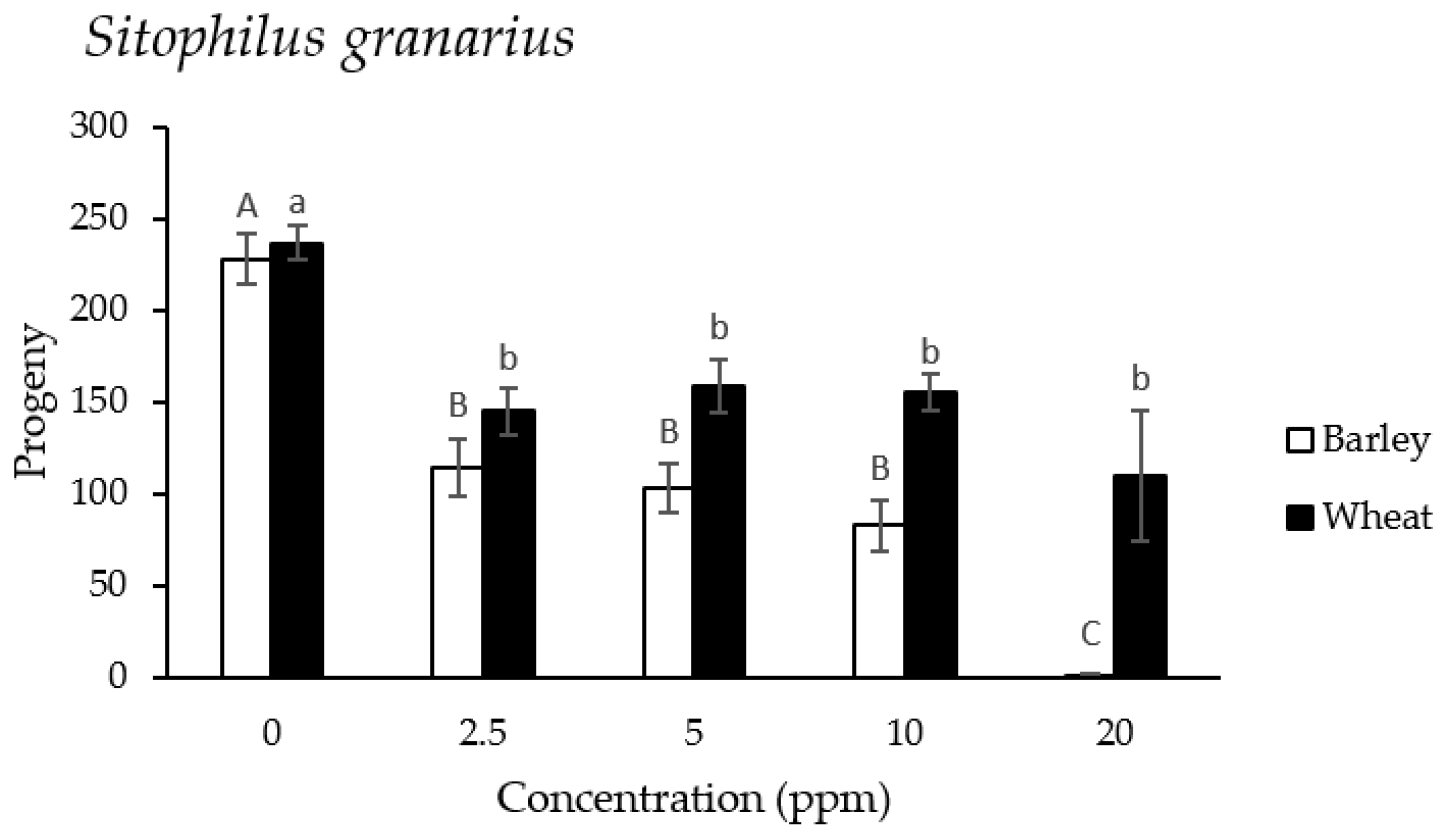
Mean progeny (± SE) of *Sitophilus granarius* after 65 days of exposure to barley or wheat treated with five concentrations (0, 2.5, 5, 10 and 20 ppm) of an azamethiphos formulation (Teenox^®^). Different uppercase letters among the concentrations within barley and different lowercase letters among the concentrations within wheat commodity indicate significant differences according to Tukey’s HSD at α < 0.05.

For *O. surinamensis*, progeny production was significantly reduced with increasing concentration in barley, whereas no significant differences among concentrations were observed in wheat (Figure 4). In contrast, for *R. dominica*, progeny production was significantly affected by concentration in both commodities, with higher concentrations resulting in lower progeny production (Figure 5). For *S. granarius*, progeny production also decreased with increasing concentrations in both commodities, with significant differences among concentrations observed for both barley and wheat (Figure 6).

### 3.3. Surface Treatments

Mortality on treated surfaces differed significantly by surface type and concentration, within each insect species (Table 3). Additionally, significant differences were recorded between surface and concentration and among the interaction of surface, and concentration, within each insect species (Table 3).

Exposure interval led to significant differences on mortality (F = 1212.3; *df* = 3; *p* < 0.0001) with all intervals involving time being also significant (*p* < 0.0001), indicating that mortality increased over time and varied depending on species, surface, and concentration.

**Figure 7:**
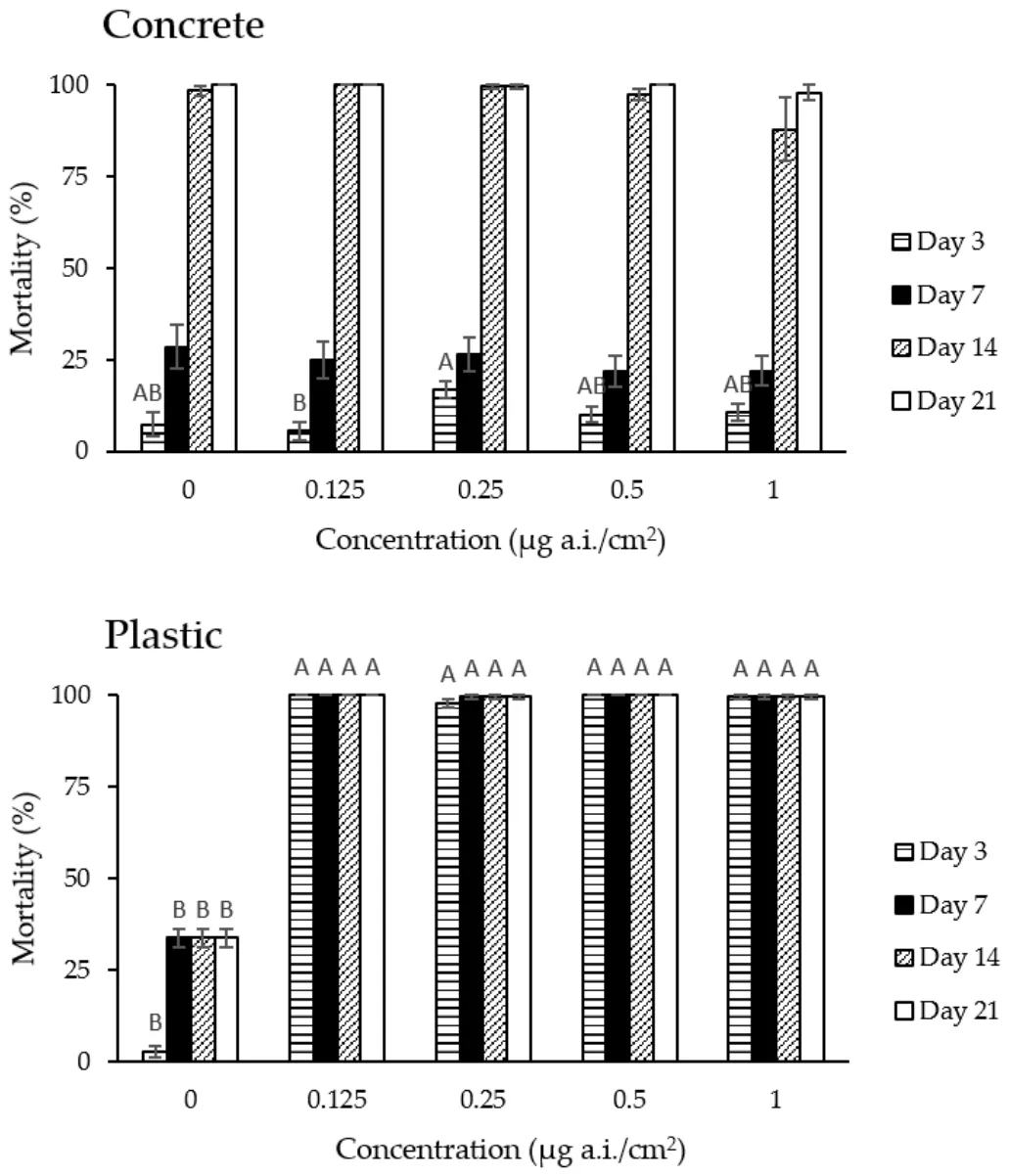
Mean mortality (% ± SE) of *Oryzaephilus surinamensis* after 3, 7, 14 and 21 days of exposure to concrete (**upper graph**) or plastic (**lower graph**) surfaces treated with five concentrations (0.125, 0.25, 0.5 and 1 μg a.i./cm^2^) of an azamethiphos formulation (Teenox^®^). Different letters among the concentrations within each day of exposure indicate significant differences according to Tukey’s HSD at α < 0.05.

For *O. surinamensis*, mortality on plastic surfaces reached almost 100% at all azamethiphos concentrations from the first interval, whereas mortality in the untreated control remained lower, approximately 3% after 3 days and about 33% after 7–21 days (Figure 7). On concrete surfaces, mortality increased with exposure time, reaching approximately 90–100% after 14 and 21 days.

**Figure 8:**
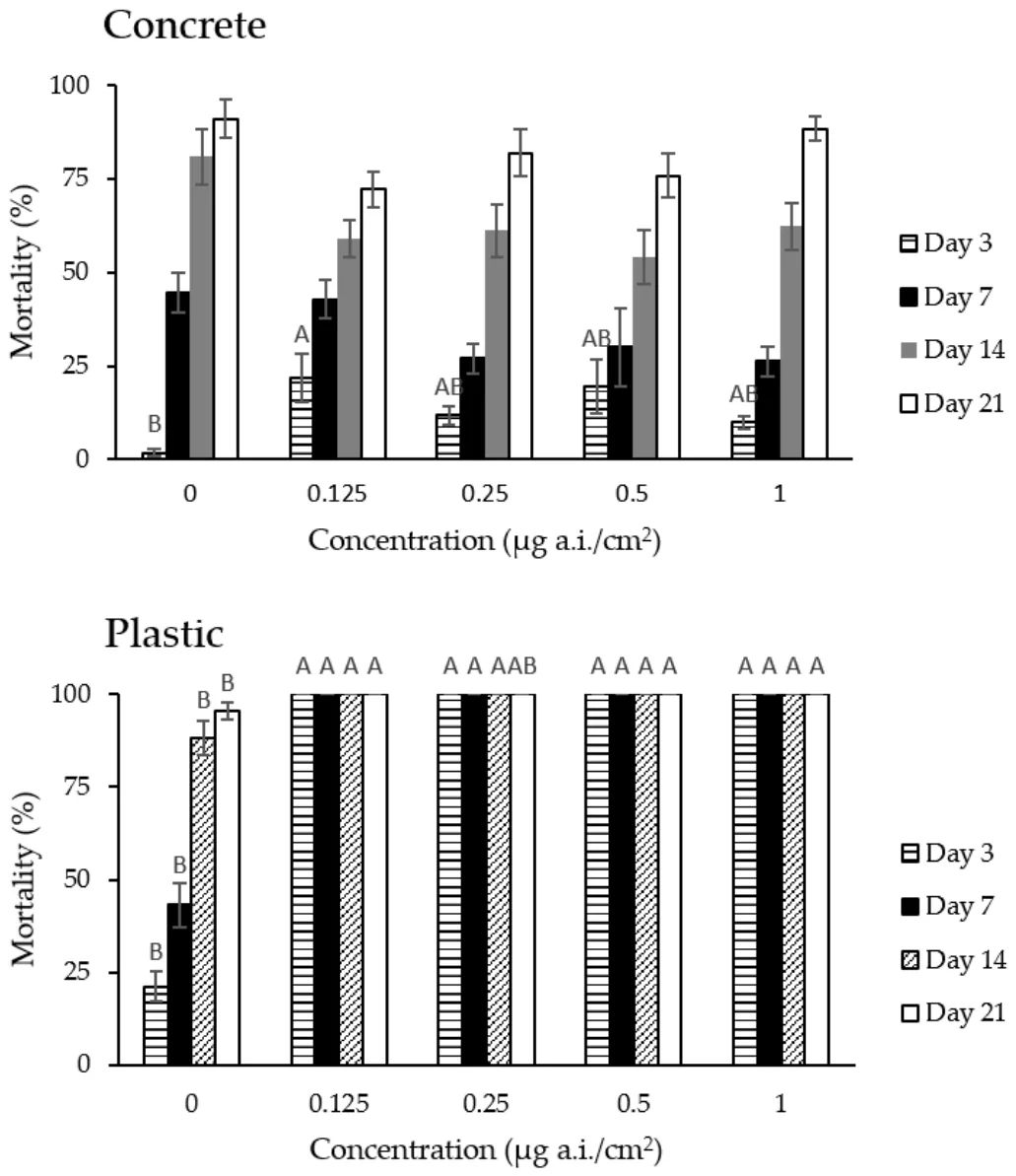
Mean mortality (% ± SE) of *Rhyzopertha dominica* after 3, 7, 14, and 21 days of exposure to concrete (**upper graph**) or plastic (**lower graph**) surfaces treated with five concentrations (0.125, 0.25, 0.5 and 1 μg a.i./cm^2^) of an azamethiphos formulation (Teenox^®^). Different letters among the concentrations within each day of exposure indicate significant differences according to Tukey’s HSD at α < 0.05.

For *R. dominica*, mortality on plastic surfaces was also very high, reaching almost 100% at all azamethiphos concentrations from the first interval (Figure 8). On concrete surfaces, mortality increased progressively with exposure interval, with the highest values recorded after 21 days of exposure. Overall, the surface-treatment results show that mortality was strongly affected by surface type, concentration and exposure duration, with azamethiphos producing high mortality, particularly on plastic surfaces and after longer exposure intervals.

## 4. Discussion

The results of the present study demonstrate that the efficacy of azamethiphos is significantly influenced by insect species, commodity type, surface type, and exposure time. Mortality increased consistently with both concentration and exposure time across all tested species, confirming the concentration-dependent and time-dependent activity of azamethiphos.

*Sitophilus granarius* was the most susceptible species, reaching high mortality levels even at 5 ppm in grain treatments, whereas *R. dominica* showed a more variable response, particularly at lower concentrations. *Oryzaephilus surinamensis* exhibited intermediate susceptibility, with mortality increasing progressively over time. These differences are consistent with previous studies indicating that susceptibility to contact insecticides varies considerably among stored-product beetle species, depending on their biology and behavior [31,32]. For example, Tsaganou et al. [32] reported marked differences in the relative susceptibility of several stored-product beetle species to thiamethoxam, with *O. surinamensis* and the confused flour beetle, *Tribolium confusum* Jancquelin du Val (Coleoptera: Tenebrionidae), generally ranking among the less susceptible species compared with bostrichid and curculionid beetles. Although the ranking of species may differ among active ingredients, these findings support the view that insecticidal efficacy is strongly species-dependent.

Commodity type also significantly affected the efficacy of azamethiphos, with higher mortality generally recorded in barley than in wheat. Similar findings have been reported in earlier studies, where the type of grain influenced insecticide efficacy, likely due to differences in kernel structure and the distribution of insecticide residues on the grain surface. For instance, Rumbos et al. [33] reported that survival of *S. oryzae* adults was significantly greater in pirimiphos-methyl-treated rice than in wheat and maize, while *T. confusum* exhibited significantly higher mortality in maize compared with the other grains. These differences may be related to variations in kernel morphology and surface characteristics among commodities, which can influence insecticide deposition, and insect contact with treated grains. These results indicate that the effectiveness of azamethiphos as a grain protectant may vary depending on the treated commodity and should be evaluated accordingly. In surface treatments, surface type had a strong effect on mortality, with higher efficacy observed on concrete compared to plastic surfaces. This finding is in line with previous research showing that surface type can influence the residual activity of insecticides, although the direction and magnitude of this effect may vary depending on the active ingredient and formulation [27,34,35]. For example, Lampiri et al. [27] reported that surface type effect was significant for all tested species except *T. confusum*, highlighting species-specific responses to treated substrates. The type of surface on which insecticides are applied plays an important role in determining their insecticidal efficacy. Several studies on surface treatments have shown that porous surfaces may reduce the effectiveness of insecticides compared with non-porous surfaces [27,29,35,36]. For instance, concrete surfaces reduced the efficacy of deltamethrin, whereas ceramic surfaces reduced the efficacy of spinosad [27]. However, in certain cases, insecticides exhibited greater efficacy on concrete compared to other surfaces [35,37], suggesting that insecticidal performance is case-specific and depends on the particular interaction between each insecticide and surface type. In the present study, the increased efficacy observed on concrete suggests that this surface may enhance insecticide availability or insect exposure under the tested conditions. This may explain why, in the present study, azamethiphos showed higher efficacy on concrete than on plastic surfaces.

Exposure duration was a key factor affecting mortality in both grain and surface treatments, as mortality levels increased significantly over time. This highlights the importance of sufficient exposure intervals for achieving maximum efficacy, particularly at lower concentrations. In contrast to grain treatments, interspecific differences were less pronounced in surface applications, indicating that surface exposure may reduce variability among species. Furthermore, progeny production was significantly reduced with increasing concentration, demonstrating that azamethiphos not only affects adult survival but also suppresses population growth. This effect is particularly important in stored-product protection, where long-term control depends on limiting reproduction in addition to causing immediate mortality.

Overall, the present findings indicate that the tested azamethiphos formulation showed promising efficacy for the control of major stored-product beetle species. However, its efficacy is dependent on multiple interacting factors, including insect species, commodity, surface type, and exposure duration. Therefore, these parameters should be considered when applying this formulation in practical storage conditions, particularly within the framework of integrated pest management (IPM) programs. Thus, practical use of this formulation should consider the target insect species, the treated commodity or surface, and the expected exposure period.

## Figures and Tables

**Table 1:** Repeated-measures MANOVA parameters for main effect (commodity and concentration) and associated interactions for mortality levels of *Oryzaephilus surinamensis*, *Rhyzopertha dominica* and *Sitophilus granarius* exposed to wheat or barley treated with an azamethiphos formulation, applied at four concentrations, i.e., 2.5, 5, 10 and 20 ppm (for each species, error *df* = 80).

Insect Species	*Sitophilus granarius*			*Rhyzopertha dominica*			*Oryzaephilus surinamensis*		
Source	F	*df*	*p*	F	*df*	*p*	F	*df*	*p*
Between interactions	214.6	9	<0.0001	16.1	9	<0.0001	162.1	9	<0.0001
Intercept	7766.5	1	<0.0001	9532.7	1	<0.0001	6965.5	1	<0.0001
Commodity	79.7	1	<0.0001	72.5	1	<0.0001	80.4	1	<0.0001
Concentration	435.3	4	<0.0001	294.2	4	<0.0001	322.8	4	<0.0001
Commodity × Concentration	27.7	4	<0.0001	10.4	4	<0.0001	21.7	4	<0.0001
Within Interactions *	4.5	27	<0.0001	28.6	27	<0.0001	7.4	27	<0.0001
Time	8.7	3	<0.0001	279.5	3	<0.0001	106.9	3	<0.0001
Time × Commodity	2.8	3	0.04	109.5	3	<0.0001	12.3	3	<0.0001
Time × Concentration *	5.6	12	<0.0001	29.8	12	<0.0001	12.8	12	<0.0001
Time × Commodity × Concentration *	3.9	12	<0.0001	17.9	12	<0.0001	2.07	12	<0.0001

* Wilks’ Lambda approximate F value.

**Table 2:** Negative binomial GLM parameters for the main effects (insect species, commodity and concentration) and associated interactions on progeny production of *Oryzaephilus surinamensis*, *Rhyzopertha dominica* and *Sitophilus granarius* at 0, 2.5, 5, 10 and 20 ppm of Teenox^®^ (Generalized x^2^/ DF = 0.90 for *Oryzaephilus surinamensis*, Generalized x^2^/DF = 1.04 for *Rhyzopertha dominica* and Generalized x^2^/DF = 0.96 for *Sitophilus granarius*).

Insect Species	*Oryzaephilus surinamensis*			*Rhyzopertha dominica*			*Sitophilus granarius*		
Source	*df*	F	*p*	*df*	F	*p*	*df*	F	*p*
Commodity	1	0.02	0.87	1	30.7	<0.0001	1	114.2	<0.0001
Concentration	4	3.03	0.02	4	5.44	<0.001	4	52.3	<0.0001
Commodity × Concentration	4	8.17	<0.0001	4	5.46	<0.001	4	31.6	<0.0001

**Table 3:** Repeated-measures MANOVA parameters for main effects (insect species, surface and concentrations) and associated interactions for mortality levels of *Rhyzopertha dominica* and *Oryzaephilus surinamensis* adults exposed to concrete or plastic surfaces treated with an azamethiphos formulation (Teenox^®^), applied at four concentrations, i.e., 0.125, 0.25, 0.5 and 1 μg a.i./cm^2^ (for each insect species, error *df* = 79).

Insect Species	*Oryzaephilus surinamensis*			*Rhyzopertha dominica*		
Source	F	*df*	*p*	F	*df*	*p*
Between Interactions	242.5	9	<0.0001	56.5	9	<0.0001
Intercept	17,707.0	1	<0.0001	4086.4	1	<0.0001
Surface	642.6	1	<0.0001	406.2	1	<0.0001
Concentration	178.0	4	<0.0001	7.3	4	<0.0001
Surface × Concentration	188.3	4	<0.0001	17.3	4	<0.0001
Within Interactions *	35.5	27	<0.0001	15.1	27	<0.0001
Time	1765.6	3	<0.0001	335.0	3	<0.0001
Time × Surface	1356.6	3	<0.0001	147.3	3	<0.0001
Time × Concentration *	8.8	12	<0.0001	13.9	12	<0.0001
Time × Surface × Concentration *	6.04	12	<0.0001	5.3	12	<0.0001

* Wilks’ Lambda approximate F value.

## Data Availability

The data supporting the findings of this study are available from the corresponding author upon reasonable request.
